# Supplementary material for: Energetic equivalence underpins the size structure of tree and phytoplankton communities
Source: Nat Commun. 2019 Jan 16;10:255. doi: 10.1038/s41467-018-08039-3 (PMC6335468; doi:10.1038/s41467-018-08039-3)
Supplement: Supplementary file 1 — Supplementary Information [file 41467_2018_8039_MOESM1_ESM.pdf]

Energetic equivalence underpins the size structure of tree and phytoplankton communities

Perkins et al.

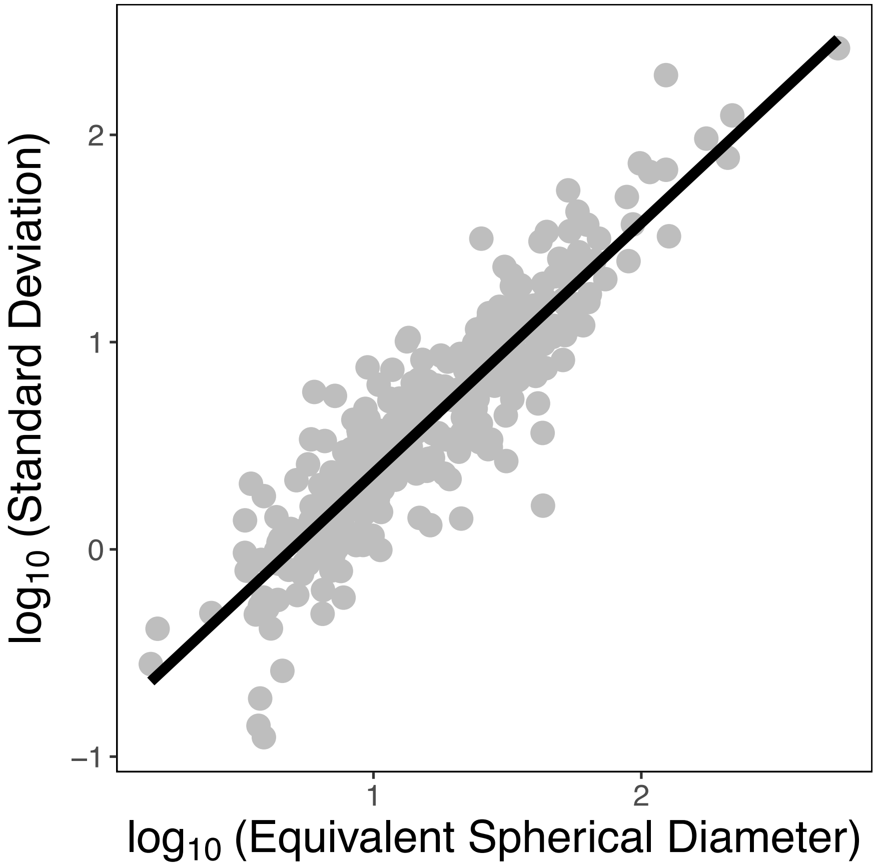

Supplementary Figure 1| Relationship between the mean and standard deviation of phytoplankton size. The strong fitted relationship derived for the size variation (Equivalent Spherical Diameter, ESD, µm) for marine and freshwater species ($SD[ESD] = 0.144\ ESD^{1.21}$, conditional $R^2 = 0.81$), allows the continuous individual size distribution to be estimated for phytoplankton datasets, where individual size measurements were not available. This was achieved by randomly sampling individual sizes for each species from a normal distribution with the reported taxon-specific ESD in the original data sources and the standard deviation estimated from the derived relationship.

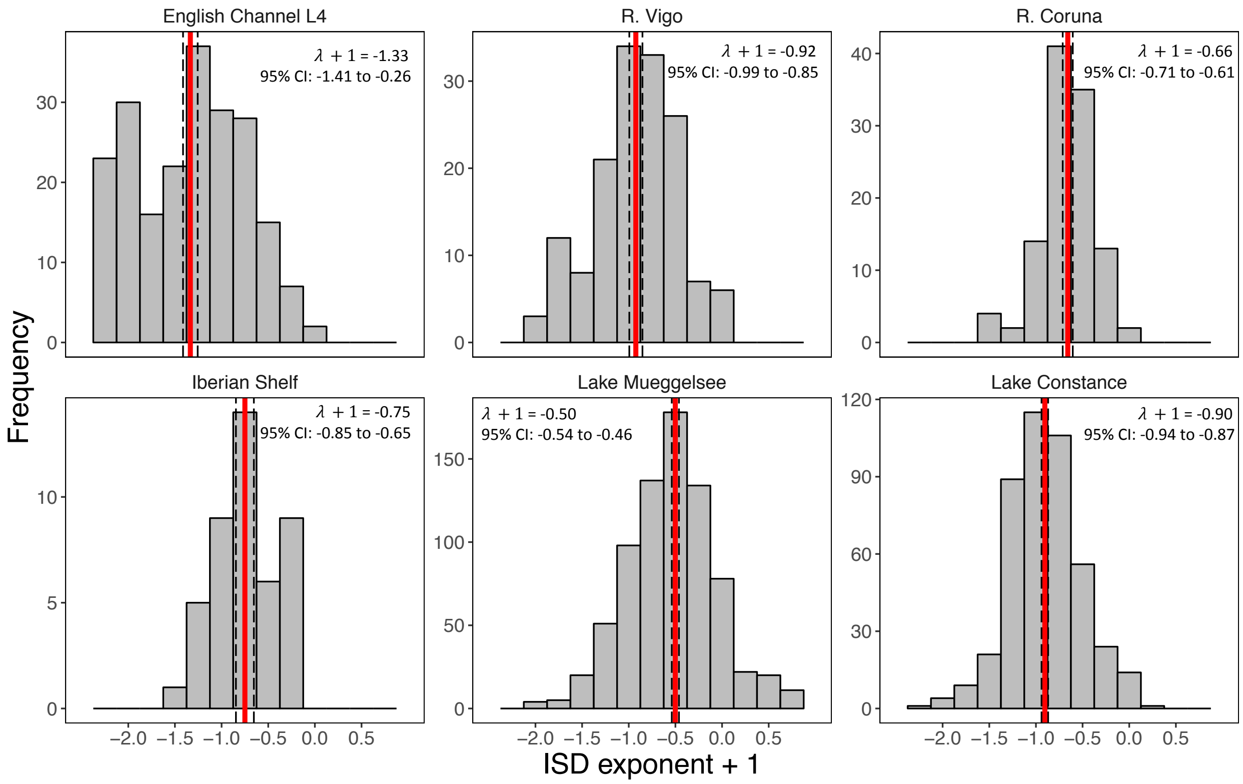

Supplementary Figure 2| Frequency histogram of individual size distribution exponents from point measurements of phytoplankton size structure. The mean among-site ISD exponent for the six stations ($\bar{\lambda} + 1$ =-0.85; 95% confidence interval: -1.06 to -0.63) does not differ from the mean among-site ISD exponent after temporal aggregation (see Results in main text) or ISD exponents from spatially distributed communities (two-sample t-test: t = -0.49, df = 93, P = 0.627; Fig. 2b). Thus, the stations included in the temporal data set can be considered representative of the stations in the spatial data set and the general form of the ISD is invariant with temporal scale. The mean within-site exponent and 95 % confidence intervals are indicated in each panel by the solid and dashed vertical lines, respectively.

Supplementary Table 1| Details of the datasets complied in this study.

Dataset	Description	Region	Number of sites	Spatial or temporal survey	Number of surveys	Sampling area or sampling depth	Survey year(s)	References
Gentry	Tropical and temperate closed-canopy forest communities	Africa (15), Asia (18), Australasia (1), Europe (4), Mesoamerica (24), North America (23), South America (102)	187	S (transect)	187	0.1 ha	1970's-1990's	[1,2]
Western Ghats	Wet evergreen / moist / dry deciduous forests	Asia	34	S (plot)	34	1 ha	1996-1997	[3]
La Selva	Tropical wet forest	Mesoamerica	5	S (plot)	5	2.24 ha	2009	[4,5]
Lahei	Tropical moist forest	Asia	3	S (plot)	3	1 ha	1998	[6–9]
DeWalt Bolivia forest plots	Tropical moist forest	South America	2	S (plot)	2	1 ha	N/A	[10]
Serimbu	Tropical rainforest	Asia	2	S (plot)	2	1 ha	1995*	[8,9,11,12]
Shirakami	Beech forest	Asia	2	S (plot)	2	1 ha	2006	[8,9,13]
ACA Amazon Forest Inventories	Tropical moist forest	South America	1	S (plot)	1	1 ha	2000-2001	[14]
Barro Colarado Island	Tropical moist forest	Mesoamerica	1	S (plot)	1	50 ha	2010	[15–17]
Cocoli	Tropical moist forest	Mesoamerica	1	S (plot)	1	4 ha	1998	[18–20]
Luquillo	Tropical moist forest	Mesoamerica	1	S (plot)	1	16 ha	1994-1996†	[21,22]
Oosting	Hardwood forest	North America	1	S (plot)	1	6.55 ha	1989	[23,24]
Sherman	Tropical moist forest	Mesoamerica	1	S (plot)	1	5.96 ha	1999	[17,19,20]
UCSC FERP	Mediterranean mixed evergreen forest	North America	1	S (plot)	1	6 ha	2007	[25]
AMT 1-3	North & south Atlantic Ocean	Equatorial (23), North Subtropical	75	S	75	7-160 m	1995-1997	[26]

TRYNITROP	Subtropical Atlantic Ocean	gyre (6), North Temperate (13), South Subtropical gyre (21), South Temperate (12) Equatorial (10), North Subtropical gyre (4), South Subtropical gyre (3)	17	S	17	surface	2007	[27]
Iberian Shelf	Coastal shelf	North Temperate	1	T (weekly)	44	0-70 m	2001-2002	[26]
PML L4 English Channel	Coastal shelf	North Temperate	1	T (weekly)	209	surface	1992-2007	[26,28]
Ría de Vigo	Coastal embayment	North Temperate	1	T (weekly)	150	0-20 m	1990-1992	[26]
Ría of A Coruña RADIALES	Coastal shelf	North Temperate	1	T (monthly)	111	0-5 m	1993-2002	[29,30]
Lake Constance	Deep (mesotrophic) lake	North Temperate	1	T (weekly)	440	0-20 m	1988-1998	[31,32]
Müggelsee	Shallow (eutrophic) lake	North Temperate	1	T (weekly)	774	0-4 m	1996-2014	[33]

The number of sites per region is denoted in parentheses. S and T indicate datasets used in the spatial and temporal analyses, respectively. *One plot has a more recent survey in 1998, however it lacks species ID. † Census 2 was chosen because information for multiple stems is not available in Census 3, and the unit of diameter is unclear in Census 4.

Supplementary Table 2| Power-law and related statistical distributions that were fitted to the data. For each distribution we give the basic functional form $f(x)$, the appropriate normalization constant C such that $\int_{x_{\min}}^{\infty} Cf(x) = 1$ and the limits. The function $\Gamma(a,b)$ is the upper incomplete gamma function, such that $\Gamma(a,0) = \Gamma(a)$ is the gamma function.

Probability density function $p(x) = Cf(x)$			
	$f(x)$	C	limits
Power-law	x^{λ}	$-(\lambda+1)x_{\min}^{-(\lambda+1)}$	$x_{\min} \le x \le \infty$
Power-exponential	$x^{\lambda}\,\mathrm{e}^{-\gamma x}$	$\dfrac{\gamma^{\lambda+1}}{\Gamma(\lambda+1, \gamma x_{\min})}$	$x_{\min} \le x \le \infty$
Bounded power-law	x^{λ}	$\dfrac{\lambda+1}{x_{\max}^{\lambda+1} - x_{\min}^{\lambda+1}}$ if $\lambda \ne 1$<br>$\dfrac{1}{\log(x_{\max}) - \log(x_{\min})}$ if $\lambda = 1$	$x_{\min} \le x \le x_{\max}$
Log-normal	$\dfrac{1}{x}\mathrm{e}^{-\frac{(\log x-\mu)^2}{2\sigma^2}}$	$\sqrt{\dfrac{2}{\pi\sigma^2}}\left[\mathrm{erfc}\left(\dfrac{\log(x_{\min})-\mu}{\sigma\sqrt{2}}\right)\right]^{-1}$	$x_{\min} \le x \le \infty$
Weibull	$\left(\dfrac{\beta}{\eta}\right)\left(\dfrac{x}{\eta}\right)^{(\beta-1)}\mathrm{e}^{-\left(\frac{x}{\eta}\right)^{\beta}}$	$\left[1 - \mathrm{e}^{-\left(\frac{x_{\min}}{\eta}\right)^{\beta}}\right]^{-1}$	$x_{\min} \le x \le \infty$

Supplementary Table 3| Identifying the best-fitting individual size distribution model.

Dataset	Number of sites	Power law	Bounded power law	Power exponential	Weibull	Log-normal
Trees	242	0.13	0.82	0.39	0.22	0.17
Phytoplankton	95	0.32	0.58	0.37	0.45	0.44

The proportion of occasions that a model was ranked among the best models for each form of power-law distributions (power law, bounded power law and power exponential) and alternative distributions (Weibull and log-normal) is given for each dataset.

REFERENCES

1. Phillips, O., Miller, J. S. & Hollowell, V. C. *Global patterns of plant diversity: Alwyn H. Gentry's forest transect data set*. **89,** (Missouri Botanical Press Louis, MO, 2002).
2. Morris, B. D. & White, E. P. The EcoData retriever: improving access to existing ecological data. *PLoS One* **8,** e65848 (2013).
3. Ramesh, B. R. *et al.* Forest stand structure and composition in 96 sites along environmental gradients in the central Western Ghats of India. *Ecology* **91,** 3118 (2010).
4. Baribault, T. W., Kobe, R. K. & Finley, A. O. Tropical tree growth is correlated with soil phosphorus, potassium, and calcium, though not for legumes. *Ecol. Monogr.* **82,** 189–203 (2012).
5. Baribault, T. W., Kobe, R. K. & Finley, A. O. Data from: Tropical tree growth is correlated with soil phosphorus, potassium, and calcium, though not for legumes. *Ecological Monographs* (2011). doi:10.5061/dryad.r9p70
6. Nishimura, T. B. & Suzuki, E. Allometric differentiation among tropical tree seedlings in heath and peat-swamp forests. *J. Trop. Ecol.* **17,** 667–681 (2001).
7. Nishimua, T. B., Suzuki, E., Kohyama, T. & Tsuyuzaki, S. Mortality and growth of trees in peat-swamp and heath forests in Central Kalimantan after severe drought. *Plant Ecol.* **188,** 165–177 (2007).
8. Lopez-Gonzalez, G., Lewis, S. L., Burkitt, M. & Phillips, O. L. ForestPlots. net: a web application and research tool to manage and analyse tropical forest plot data. *J. Veg. Sci.* **22,** 610–613 (2011).
9. Lopez-Gonzalez, G., Lewis, S. L., Burkitt, M. & Phillips, O. L. ForestPlots.net database. http://www.forestplots.net. Date of extraction: July 6, 2012.
10. DeWalt, S. J., Bourdy, G., De Michel, L. R. C. & Quenevo, C. Ethnobotany of the Tacana: quantitative inventories of two permanent plots of northwestern Bolivia. *Econ. Bot.* **53,** 237–260 (1999).
11. Kohyama, T., Suzuki, E., Partomihardjo, T. & Yamada, T. Dynamic steady state of patch-mosaic tree size structure of a mixed dipterocarp forest regulated by local crowding. *Ecol. Res.*

16, 85–98 (2001).

12. Kohyama, T., Suzuki, E., Partomihardjo, T., Yamada, T. & Kubo, T. Tree species differentiation in growth, recruitment and allometry in relation to maximum height in a Bornean mixed dipterocarp forest. *J. Ecol.* **91,** 797–806 (2003).
13. Nakashizuka, T. *et al.* Monitoring dynamics of beech forests with different structure in Shirakami Mountains. *Tohoku J. For. Sci.* **8,** 67–74 (2003).
14. Pitman, N. C. A., Cerón, C. E., Reyes, C. I., Thurber, M. & Arellano, J. Catastrophic natural origin of a species-poor tree community in the world's richest forest. *J. Trop. Ecol.* **21,** 559–568 (2005).
15. Hubbell, S. P. *et al.* Light-gap disturbances, recruitment limitation, and tree diversity in a neotropical forest. *Science (80-.).* **283,** 554–557 (1999).
16. Hubbell, S. P., Condit, R. & Foster, R. B. Barro Colorado Forest Census plot data. http://ctfs.arnarb.harvard.edu/webatlas /datasets/bci/. Accessed April 9, 2012. (2005).
17. Condit, R. *Tropical forest census plots: methods and results from Barro Colorado Island, Panama and a comparison with other plots*. (Springer Science & Business Media, 1998).
18. Condit, R., Sukumar, R., Hubbell, S. P. & Foster, R. B. Predicting population trends from size distributions: a direct test in a tropical tree community. *Am. Nat.* **152,** 495–509 (1998).
19. Condit, R. *et al.* Tropical forest dynamics across a rainfall gradient and the impact of an El Nino dry season. *J. Trop. Ecol.* **20,** 51–72 (2004).
20. Pyke, C. R., Condit, R., Aguilar, S. & Lao, S. Floristic composition across a climatic gradient in a neotropical lowland forest. *J. Veg. Sci.* **12,** 553–566 (2001).
21. Zimmerman, J. K. *et al.* Responses of tree species to hurricane winds in subtropical wet forest in Puerto Rico: implications for tropical tree life histories. *J. Ecol.* 911–922 (1994).
22. Thompson, J. *et al.* Land use history, environment, and tree composition in a tropical forest. *Ecol. Appl.* **12,** 1344–1363 (2002).
23. Reed, R. A., Peet, R. K., Palmer, M. W. & White, P. S. Scale dependence of vegetation-environment correlations: A case study of a North Carolina piedmont woodland. *J. Veg. Sci.* **4,** 329–340 (1993).
24. Palmer, M. W., Peet, R. K., Reed, R. A., Xi, W. & White, P. S. A multiscale study of vascular plants in a North Carolina piedmont forest. *Ecology* **88,** 2674 (2007).

25. Gilbert, G. S. *et al.* Beyond the tropics: forest structure in a temperate forest mapped plot. *J. Veg. Sci.* **21,** 388–405 (2010).

26. Cermeño, P. & Figueiras, F. G. Species richness and cell-size distribution: size structure of phytoplankton communities. *Mar. Ecol. Prog. Ser.* **357,** 79–86 (2008).

27. Huete-Ortega, M., Cermeño, P., Calvo-Díaz, A. & Marañón, E. Isometric size-scaling of metabolic rate and the size abundance distribution of phytoplankton. *Proc. R. Soc. B Biol. Sci.* **279,** 1815–1823 (2012).

28. Widdicombe, C. E., Eloire, D., Harbour, D., Harris, R. P. & Somerfield, P. J. Time series of phyto- and microzooplankton abundance and composition at station L4 in the English Channel from 1988 to 2009. *Supplement to: Widdicombe, CE et al. (2010): Long-term phytoplankton community dynamics in the Western English Channel. Journal of Plankton Research, 32(5), 643-655, https://doi.org/10.1093/plankt/fbp127* (2010). doi:10.1594/PANGAEA.758061

29. Huete-Ortega, M. *et al.* Distinct patterns in the size-scaling of abundance and metabolism in coastal and open-ocean phytoplankton communities. *Mar. Ecol. Prog. Ser.* **515,** 61–71 (2014).

30. Bode, A., Estévez, M. G., Varela, M. & Vilar, J. A. Annual trend patterns of phytoplankton species abundance belie homogeneous taxonomical group responses to climate in the NE Atlantic upwelling. *Mar. Environ. Res.* **110,** 81–91 (2015).

31. Gaedke, U. The size distribution of plankton biomass in a large lake and its seasonal variability. *Limnol. Oceanogr.* **37,** 1202–1220 (1992).

32. Gaedke, U. Functional and taxonomical properties of the phytoplankton community of large and deep Lake Constance: Interannual variability and response to re-oligotrophication (1979-1993). *Adv. Limnol. Stuttgart* 119–141 (1998).

33. Özkundakci, D., Gsell, A. S., Hintze, T., Täuscher, H. & Adrian, R. Winter severity determines functional trait composition of phytoplankton in seasonally ice-covered lakes. *Glob. Chang. Biol.* **22,** 284–298 (2016).
