# Supplementary material for: Energetic equivalence underpins the size structure of tree and phytoplankton communities
Source: Nat Commun. 2019 Jan 16;10:255. doi: 10.1038/s41467-018-08039-3 (PMC6335468; doi:10.1038/s41467-018-08039-3)
Supplement: Supplementary file 3 — Description of Additional Supplementary Files [file 41467_2018_8039_MOESM3_ESM.pdf]

Supplementary Data 1| Summary output for the fit of power-law distributions to individual size data for each tree and phytoplankton community. These data were used to generate Table 1 and Figure 2 in the manuscript.
