# Supplementary material for: Energetic equivalence underpins the size structure of tree and phytoplankton communities
Source: Nat Commun. 2019 Jan 16;10:255. doi: 10.1038/s41467-018-08039-3 (PMC6335468; doi:10.1038/s41467-018-08039-3)
Supplement: Supplementary file 5 — Reporting Summary [file 41467_2018_8039_MOESM5_ESM.pdf]

nature research

Corresponding author(s): Daniel Perkins & Gabriel Yvon-Durocher

Reporting Summary

Nature Research wishes to improve the reproducibility of the work that we publish. This form provides structure for consistency and transparency in reporting. For further information on Nature Research policies, see Authors & Referees and the Editorial Policy Checklist.

Statistical parameters

When statistical analyses are reported, confirm that the following items are present in the relevant location (e.g. figure legend, table legend, main text, or Methods section).

n/a	Confirmed	
☐	☒	The exact sample size (*n*) for each experimental group/condition, given as a discrete number and unit of measurement
☐	☒	An indication of whether measurements were taken from distinct samples or whether the same sample was measured repeatedly
☐	☒	The statistical test(s) used AND whether they are one- or two-sided<br>*Only common tests should be described solely by name; describe more complex techniques in the Methods section.*
☒	☐	A description of all covariates tested
☐	☒	A description of any assumptions or corrections, such as tests of normality and adjustment for multiple comparisons
☐	☒	A full description of the statistics including central tendency (e.g. means) or other basic estimates (e.g. regression coefficient) AND variation (e.g. standard deviation) or associated estimates of uncertainty (e.g. confidence intervals)
☐	☒	For null hypothesis testing, the test statistic (e.g. *F*, *t*, *r*) with confidence intervals, effect sizes, degrees of freedom and *P* value noted<br>*Give P values as exact values whenever suitable.*
☒	☐	For Bayesian analysis, information on the choice of priors and Markov chain Monte Carlo settings
☒	☐	For hierarchical and complex designs, identification of the appropriate level for tests and full reporting of outcomes
☒	☐	Estimates of effect sizes (e.g. Cohen's *d*, Pearson's *r*), indicating how they were calculated
☐	☒	Clearly defined error bars<br>*State explicitly what error bars represent (e.g. SD, SE, CI)*

Our web collection on statistics for biologists may be useful.

Software and code

Policy information about availability of computer code

Data collection	No software was used for data collection
Data analysis	Data analysis was performed using the the R statistical platform (v. 3.4.1)

For manuscripts utilizing custom algorithms or software that are central to the research but not yet described in published literature, software must be made available to editors/reviewers upon request. We strongly encourage code deposition in a community repository (e.g. GitHub). See the Nature Research guidelines for submitting code & software for further information.

Data

Policy information about availability of data

All manuscripts must include a data availability statement. This statement should provide the following information, where applicable:

- Accession codes, unique identifiers, or web links for publicly available datasets
- A list of figures that have associated raw data
- A description of any restrictions on data availability

The summary data used to generate Table 1 and Figure 2 are available in Supplementary Data 1. The analysis R code, as well as a subset of the analyzed data, is archived in a Figshare public repository (https://doi.org/10.6084/m9.figshare.7178000.v1). The data included in the deposit are specifically intended for the

replication of the analysis procedure. Therefore, researchers interested in using the data for purposes other than replicating our analyses are advised to obtain the raw data from the original sources cited here, as other useful information from the original data might not be included.

Field-specific reporting

Please select the best fit for your research. If you are not sure, read the appropriate sections before making your selection.

☐ Life sciences ☐ Behavioural & social sciences ☒ Ecological, evolutionary & environmental sciences

For a reference copy of the document with all sections, see nature.com/authors/policies/ReportingSummary-flat.pdf

Ecological, evolutionary & environmental sciences study design

All studies must disclose on these points even when the disclosure is negative.

Study description	This study involves the meta-analysis of individual body size distributions from a global dataset of 2062 tree and phytoplankton surveys from 242 terrestrial and 95 aquatic (freshwater and marine) sampling locations. A factorial analysis was performed testing for differences between groups (trees and phytoplankton).
Research sample	Natural tree and phytoplankton communities. Further details can be found in the original sources (full details given in Supplementary Table 1)
Sampling strategy	Sample sizes were based upon established sampling protocols outlined in the original sources (Supplementary Table 1)
Data collection	Summary of data collection given in methods and further details given in original sources (full details given in Supplementary Table 1)
Timing and spatial scale	Meta-analysis based on data collected over various temporal and spatial scales (from weekly sample collection within one location to global cruise transects). Further details on timing and spatial scales given in original sources (full details given in Supplementary Table 1)
Data exclusions	"We removed three AMT stations from the analysis (Supplementary Data 1), as these were outliers."
Reproducibility	Reproducibility is not relevant to this study, which was not an experiment but concerns surveys of natural communities.
Randomization	Randomization is not relevant to this study, which was not an experiment but concerns surveys of natural communities.
Blinding	Blinding is not relevant to this study, which was not an experiment but concerns surveys of natural communities.

Did the study involve field work? ☐ Yes ☒ No

Reporting for specific materials, systems and methods

Materials & experimental systems

n/a	Involved in the study	
☒	☐	Unique biological materials
☒	☐	Antibodies
☒	☐	Eukaryotic cell lines
☒	☐	Palaeontology
☒	☐	Animals and other organisms
☒	☐	Human research participants

Methods

n/a	Involved in the study	
☒	☐	ChIP-seq
☒	☐	Flow cytometry
☒	☐	MRI-based neuroimaging
